# Sustainable Surfactin Production by *Bacillus subtilis* Using Crude Glycerol from Different Wastes

**DOI:** 10.3390/molecules26123488

**Published:** 2021-06-08

**Authors:** Tomasz Janek, Eduardo J. Gudiña, Xymena Połomska, Piotr Biniarz, Dominika Jama, Lígia R. Rodrigues, Waldemar Rymowicz, Zbigniew Lazar

**Affiliations:** 1 Department of Biotechnology and Food Microbiology, Wrocław University of Environmental and Life Sciences, 51-630 Wrocław, Poland; xymena.polomska@upwr.edu.pl (X.P.); piotr.biniarz@upwr.edu.pl (P.B.); 109215@student.upwr.edu.pl (D.J.); waldemar.rymowicz@upwr.edu.pl (W.R.); zbigniew.lazar@upwr.edu.pl (Z.L.); 2 Centre of Biological Engineering, University of Minho, 4710-057 Braga, Portugal; egudina@deb.uminho.pt (E.J.G.); lrmr@deb.uminho.pt (L.R.R.); 3 Łukasiewicz Research Network—PORT Polish Center for Technology Development, 54-066 Wrocław, Poland

**Keywords:** *Bacillus subtilis*, biosurfactant, surfactin, lipopeptides, industrial wastes, crude glycerol

## Abstract

Most biosurfactants are obtained using costly culture media and purification processes, which limits their wider industrial use. Sustainability of their production processes can be achieved, in part, by using cheap substrates found among agricultural and food wastes or byproducts. In the present study, crude glycerol, a raw material obtained from several industrial processes, was evaluated as a potential low-cost carbon source to reduce the costs of surfactin production by *Bacillus subtilis* #309. The culture medium containing soap-derived waste glycerol led to the best surfactin production, reaching about 2.8 g/L. To the best of our knowledge, this is the first report describing surfactin production by *B. subtilis* using stearin and soap wastes as carbon sources. A complete chemical characterization of surfactin analogs produced from the different waste glycerol samples was performed by liquid chromatography–mass spectrometry (LC-MS) and Fourier transform infrared spectroscopy (FTIR). Furthermore, the surfactin produced in the study exhibited good stability in a wide range of pH, salinity and temperatures, suggesting its potential for several applications in biotechnology.

## 1. Introduction

Surfactants are amphiphilic molecules containing at least one hydrophobic and one hydrophilic domain. Such structure enables their location at the interface between fluids of different polarities, reducing surface and interfacial tension [1]. Surfactants are commonly used as cleaning and washing agents, but they can also be applied as dispersants, moisturizers, emulsifiers, anti-caking and foaming agents in many sectors, including cosmetics, food, paper, textiles, petroleum and other industries [2]. They are also used to combat microbes, viruses, pests and weeds, as well as in the bioremediation of petroleum-contaminated environments [3,4]. Furthermore, several studies have shown that the selected surfactants in pharmaceutical formulations can improve the effectiveness of anticancer drugs [5]. Due to their great variety of applications, surfactants are produced on a large scale. It is estimated that the annual production of these compounds is about 16 million tons [6]. Most surfactants are obtained via chemical synthesis using petroleum-based compounds as precursors [7]. However, for the sake of environmental protection and consumer health, naturally occurring biosurfactants are more attractive due to their lower toxicity and higher biodegradability compared with synthetic surfactants [8].

Nowadays, a considerable number of these “green” biomolecules produced by microorganisms and plants are known. They include glycolipids and phospholipids, lipopeptides and lipoproteins, fatty acids as well as surfactants with a polymeric structure [9,10]. Bacteria of the *Bacillus* genus mainly produce lipopeptide biosurfactants. The best-known biosurfactant, surfactin, is a cyclic lipopeptide composed of a seven-amino-acid hydrophilic peptide ring and a hydrophobic chain of β-hydroxy fatty acid with a length of 12–16 carbon atoms [11]. The biosynthesis of surfactin is carried out by multi-domain-specific non-ribosomal peptide synthetases (NRPSs). *Bacillus subtilis*, *Bacillus amyloliquefaciens* and *Bacillus licheniformis* are the main producers of this compound [12]. Surfactin is the most powerful biosurfactant discovered so far. It can reduce the surface tension of water from 72 to 27 mN/m at a critical micelle concentration (CMC) of 20 mg/L, thereby displaying strong emulsifying and foaming activities [13]. Due to its ability to disintegrate phospholipidic membranes, surfactin exhibits strong antibacterial, antifungal, antiviral and anticancer activities [14,15,16,17]. In addition, it can prevent the formation of or even destroy existing bacterial biofilms [18,19].

Due to its properties, surfactin has a wide variety of potential applications [20,21]. However, a significant barrier to its use is its low production yield, resulting in high production costs [22]. To overcome this limitation, many researchers are using genetic engineering in an attempt to enhance surfactin production [9]. In addition, attempts at the optimization of media composition and operational conditions were conducted to increase production [23]. In order to reduce the production costs, cheap substrates are sought, most often among agricultural and food wastes or byproducts [24]. These substrates are usually rich in carbon and organic nitrogen sources, vitamins and minerals [25]. The raw materials most commonly used are wastes rich in starch, sucrose or other sugars (cassava wastewater, corn steep liquor, potato peels, wheat bran and beet molasses) [20] as well as vegetable oils or wastes resulting from their production (olive mill wastewater, rice bran oil and sunflower oil) [26,27]. Furthermore, several studies have reported the possibility of producing surfactin by solid-state fermentation using wastes that are difficult to hydrolyze, such as rice straw, alone or in combination with other food byproducts [28]. Crude glycerol from biodiesel and other oleo-chemical production processes can also be used as a cheap component of the culture medium, namely as the water-soluble carbon source [29]. The use of waste glycerol in several biotechnological processes is becoming very important due to the large amounts that are being generated by the biodiesel industry every year. Thus, its use in the biosurfactant production process offers an opportunity to reduce production costs. The detailed glycerol utilization pathway and surfactin biosynthesis in *Bacillus* strains were described by Zhou et al. [30]. The transcription levels of genes encoding proteins from the glycerol utilization pathway and modular surfactin synthase were relatively high; therefore, *Bacillus* strains can efficiently use glycerol to produce surfactin.

The previously reported *B. subtilis* #309 could produce different surfactin congeners using culture media containing various pure carbon and nitrogen sources [21]. Among the surfactants produced by *B. subtilis* #309, the most abundant were the surfactin-C_13_, surfactin-C_14_ and surfactin-C_15_ homologs. The aim of this study was to evaluate the production of lipopeptide biosurfactants by *B. subtilis* #309 using low-cost culture media, formulated using waste glycerol from different sources, namely from biodiesel-, stearin- and soap-production processes. Additionally, the molecular structure and physicochemical properties (including CMC) and the stability at different temperatures, pH values and NaCl concentrations of the produced surfactants were studied.

## 2. Results and Discussion

### 2.1. Evaluation of Crude Glycerol from Different Sources as a Substrate for Biosurfactant Production

The carbon source present in the culture medium is one of the most important factors in the production of biosurfactants [31,32]. Waste glycerol has been widely used as a substrate in several bioprocesses [33,34,35]. The utilization of low-cost carbon sources, such as waste glycerol, is an interesting alternative for lipopeptide biosurfactant biosynthesis by microorganisms. In this work, waste glycerol originating from four different industries was evaluated as a low-cost carbon source for the production of lipopeptide biosurfactants by *B. subtilis* #309. The origin of crude glycerol was as follows: G1 and G2 derived from biodiesel production, G3 derived from stearin production, and G4 derived from soap production. Pure glycerol (G5) was used as a reference carbon source. The composition of the raw glycerol provides important data to aid the culture medium formulation. It required nitrogen supplementation, as this nutrient was present at low concentrations in the crude glycerol samples (Table S1).

**Figure 1:**
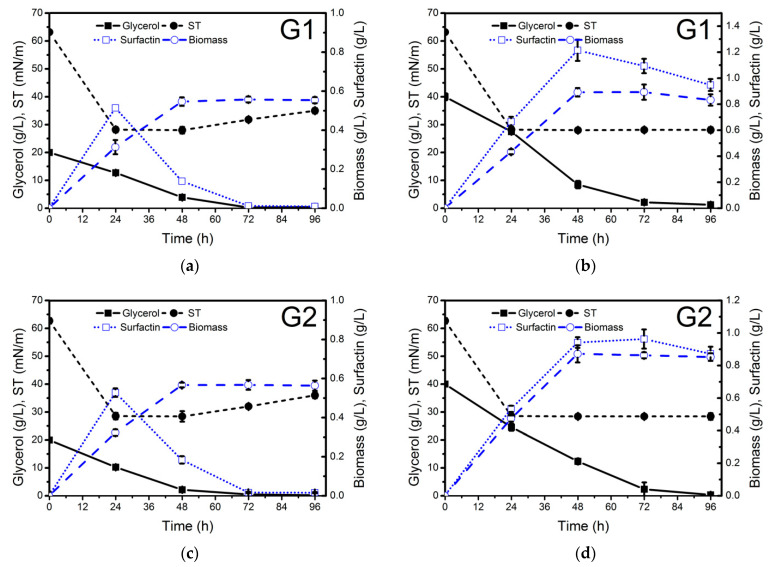

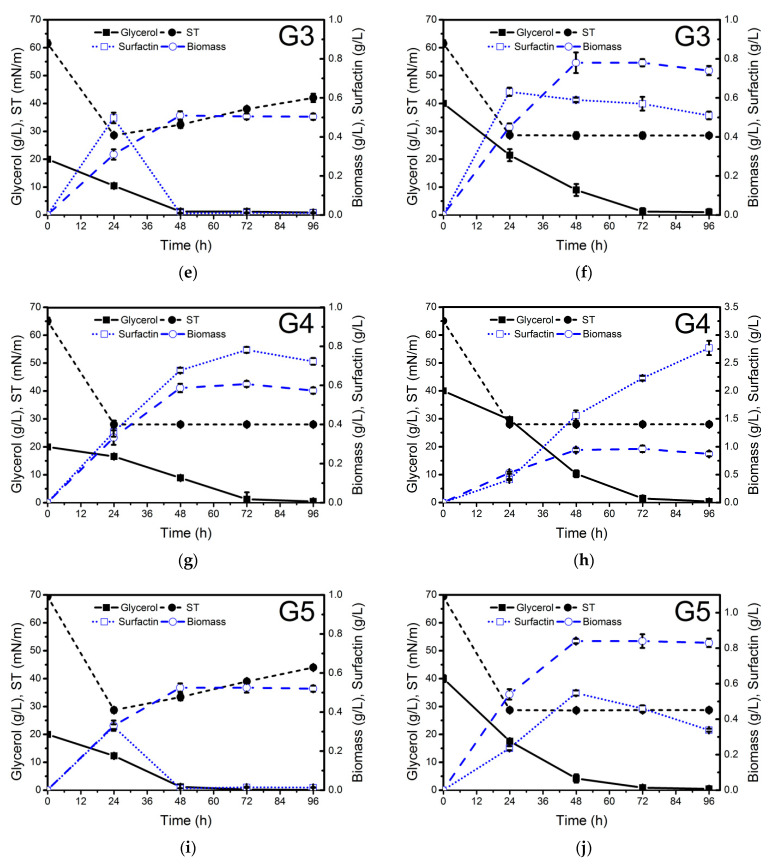
Glycerol consumption (g/L), biomass (g dry weight/L), surface tension (ST, mN/m) and surfactin production (g/L) by *Bacillus subtilis* #309 grown in a mineral salt medium (MSM) supplemented with 20 g/L (**a**,**c**,**e**,**g**,**i**) and 40 g/L (**b**,**d**,**f**,**h**,**j**) of waste glycerol (G1–G4) from different sources and pure glycerol (G5). The cultures were performed at 37 °C and 160 rpm for 96 h. The biomass concentration was determined gravimetrically, i.e., the biomass dry weight (or total solids) per liter was measured using a balance. The results represent the mean ± standard deviation of three independent experiments performed in triplicate.

The different waste glycerol samples were evaluated for biosurfactant production at two different concentrations: 20 g/L (C/N ratio 5.5) and 40 g/L (C/N ratio 11) (Figure 1). NH_4_NO_3_ (4 g/L) was used as a nitrogen source. The highest surfactin production (2.8 g/L) was achieved after 96 h of growth when soap-derived waste glycerol (G4) (Figure 1h) was used as a carbon source. Lower surfactin concentration was obtained using raw glycerol derived from stearin (G3) production (0.41 g/L), similar to that achieved with pure glycerol (0.33 g/L). These results are in agreement with the surface tension values measured in the cell-free supernatants obtained from the different culture media throughout the whole process (Figure 1). The differences observed in surfactin production using the different raw glycerol samples may be due to their different composition.

As can be observed in Figure 1h, when soap-derived waste glycerol was used as a carbon source, not only was higher surfactin production observed but also higher biomass concentration was achieved in a medium with a C/N ratio of 11 (0.96 g cell dry weight/L) compared to a medium with a C/N ratio of 5.5 (0.6 g cell dry weight/L). In both cases, glycerol was exhausted between 48 and 72 h of growth. Although in both processes, surfactin production was growth-associated, in the culture medium with a C/N ratio of 11, considerable production was also observed during the stationary phase (Figure 1). After glycerol depletion, surfactin was quickly utilized by the bacteria as a carbon source (Figure 1a,e,i). Therefore, soap-derived waste glycerol can be considered a promising low-cost carbon source to reduce the production costs of surfactin. Biosurfactant production by *B. subtilis* using pure and waste glycerol derived from biodiesel production as a carbon source has been studied by several authors. The amount of surfactin produced ranged from 0.20 to 1.38 g/L [21,36,37,38,39]. However, this is the first report dealing with surfactin production using soap-derived waste glycerol.

The surface tension (ST) of the cell-free supernatants obtained from the different cultures was determined using the Du Noüy ring method. The lowest ST values were obtained when soap-derived waste glycerol was used as a carbon source (Figure 1i). The ST was reduced to 28.0 mN/m after 24 h, and the ST of 10 and 100 times diluted (ST^−1^, ST^−2^) cell-free supernatants at 96 h was 28.5 and 36.4 mN/m (Table 1), respectively, thus indicating that the surfactin concentration in the cell-free supernatant was at least 10 times its CMC value of 15 mg/L at an ST of 30 mN/m (as shown in paragraph 2.4). The use of biodiesel-derived waste glycerol G1 as a carbon source resulted in a lower ST^−1^ value (29.1 mN/m) compared to G2 (30.7 mN/m) (Table 1). The stearin-derived waste glycerol also offered good results regarding ST reduction (ST^−1^ = 31.6 mN/m). These ST values are similar to several other reports for lipopeptide biosurfactant production by *Bacillus* strains [21].

### 2.2. Chromatographic Characterization of Biosurfactants Produced by B. subtilis #309 Using Glycerol from Different Sources

The biosurfactants produced by *B. subtilis* #309 grown in a mineral salt medium (MSM) supplemented with raw glycerol from different sources and pure glycerol was characterized through liquid chromatography–mass spectrometry (LC-MS). The relative amounts of the different surfactin variants were calculated according to the area of the peaks identified in each sample, considering the sum of the areas of all the peaks detected as 100% (Figure S1). Subsequently, each peak was identified according to the extracted ion chromatograms. As shown in Table 2, the surfactin mixtures produced in the different culture media contained five homologs with mass–charge ratios (*m/z*) of 994.64, 1008.66, 1022.68, 1036.69 and 1050.71. The same homologs were identified in the surfactin standard.

The possible structures of surfactin variants were elucidated on the basis of collected MS/MS spectra, as described by Moro et al. [40] and Ma et al. [41]. The MS/MS spectra are shown in Supplementary Materials (Figures S2–S6). The composition of the culture medium can affect not only the amount but also the type of biosurfactant produced by a particular microorganism. As can be seen in Table S3, regardless of the glycerol used, *B. subtilis* #309 produced six surfactin analogs (C_12_, C_13_, C_14_, C_15_, C_16_ and C_17_ surfactin). However, some differences could be observed in the relative abundance of each analog depending on the substrate. In all the cases, the most abundant analog was C_14_ surfactin (relative abundance between 42.0 and 54.8%), followed by C_15_ surfactin (25.2–34.7%) and C_13_ surfactin (8.9–15.8%). The same analogs were detected in the surfactin standard, although in that case, C_15_ surfactin (41.8%) was more abundant than C_14_ surfactin (35.9%) (Table S3). C_13_, C_14_ and C_15_ surfactants are usually the predominant analogs identified in surfactin mixtures produced by different *B. subtilis* isolates [42]. The amino acid sequences of surfactants produced by *B. subtilis* #309 were similar to those present in the surfactin standard (Figures S2–S6). These results indicate that the chemical structure of surfactants produced by *B. subtilis* #309 is very similar to that of other surfactin mixtures produced by different *Bacillus* strains [43,44,45].

The surfactin produced by *B. subtilis* #309 and the surfactin standard (Merck) were subjected to thin-layer chromatography (TLC) to characterize their homogeneity. The TLC showed single spots with bromothymol blue as the staining reagent (Figure S7). Both compounds have a similar Rf value of ~0.67 and hence are highly likely to have a very similar structure.

### 2.3. Evaluation of Emulsifying Properties of the Biosurfactant Produced Using Culture Medium with Glycerol from Different Sources

**Figure 2:**
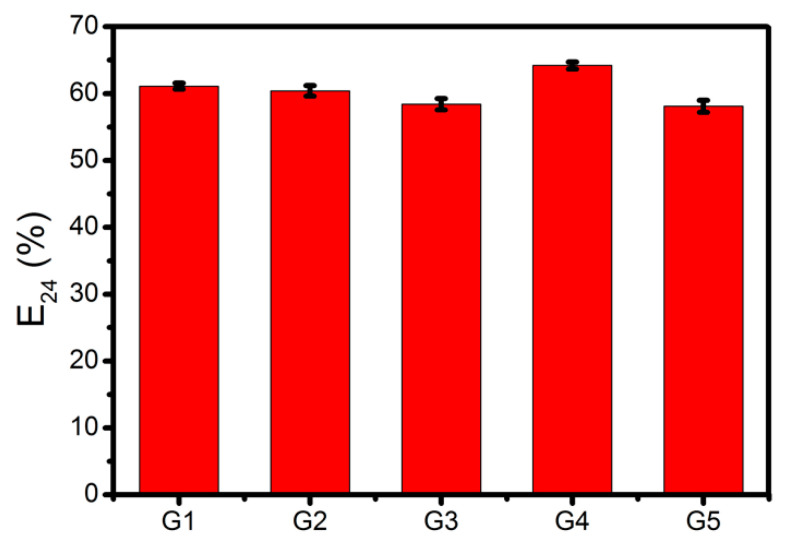
Emulsifying activity (E_24_, %) against n-hexadecane of cell-free supernatants obtained from cultures of *Bacillus subtilis* #309 grown in mineral salt medium (MSM) supplemented with glycerol from different sources (G1, G2, G3, G4 or G5). The results represent the mean ± standard deviation of three independent experiments performed in triplicate.

The emulsifying activity of the surfactin mixtures produced by *B. subtilis* #309 using pure glycerol and the different waste glycerol samples as the carbon source was evaluated using the cell-free supernatants obtained after 96 h of growth. As can be seen in Figure 2, high emulsification indexes (E_24_) (between 58% and 64%) were obtained when mixing the culture supernatants with n-hexadecane, regardless of the carbon source used. The emulsifying activity of the biosurfactants produced by *B. subtilis* #309 was similar to others also produced by *Bacillus* strains [21]. These results suggest that the surfactin-containing culture supernatants can potentially be used as effective emulsifiers in enhanced oil recovery and bioremediation.

### 2.4. Determination of Critical Micelle Concentration (CMC)

**Figure 3:**
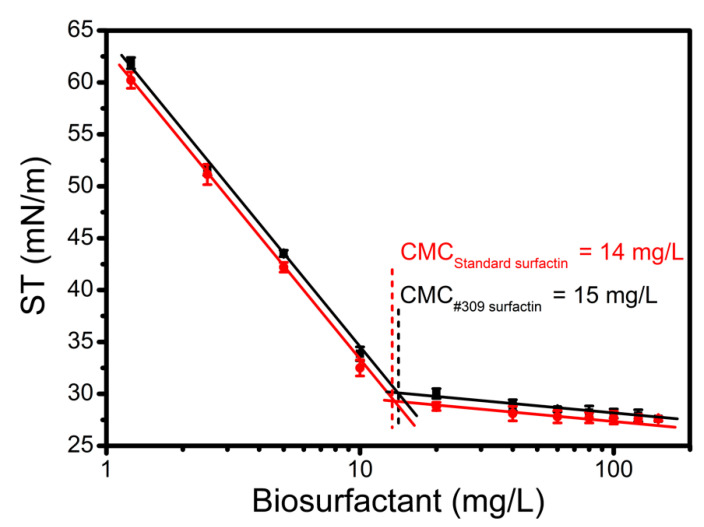
Surface tension (ST) versus logarithm of surfactin concentration. The reference surface tension value (HEPES buffer) was 70.4 mN/m. The results represent the mean ± standard deviation of three independent experiments performed in triplicate.

The CMC is an important physicochemical property of surfactants and microbial biosurfactants, as it is an indicator of their efficiency. The CMC of surfactin produced by *B. subtilis* #309 using soap-derived crude glycerol (G4) as a carbon source was determined by measuring the ST of surfactin solutions in HEPES buffer at different concentrations (1.25–150 mg/L). The ST decreased with the increase of surfactin concentration, as expected (Figure 3). The ST of HEPES buffer was reduced from 71 to 28 mN/m. The CMC value could be determined from the breaking point of ST versus the logarithm of surfactin concentration (Figure 3). The CMCs of the surfactin standard and the surfactin produced by *B. subtilis* #309 were obtained from the graph and were found to be 14 mg/L and 15 mg/L, respectively. These results are in good agreement with previous reports on surfactin isolated from other *B. subtilis* strains (CMCs between 10 and 28 mg/L) [38,42,45]. Using Equation (1) [46], the purity of the test surfactin was found to be about 93%.

(1) $$\%\;\mathrm{impurities}=\left[\left(\frac{CMC_{test}}{CMC_{std}}\right)-1\right]\times 100\%$$

### 2.5. Effect of pH, Temperature and Salinity on Biosurfactant Activity

Biosurfactants can potentially be used in numerous industrial fields; however, for some applications, they must be stable in a wide range of pH values, salinities and temperatures [47]. For example, for application in detergent formulations or enhanced oil recovery, there are still some challenges regarding the stability of biosurfactants over time at extreme pH values and temperatures [48]. For that reason, the stability of the biosurfactant produced by *B. subtilis* #309 using soap-derived crude glycerol was evaluated in different environmental conditions.

**Figure 4:**
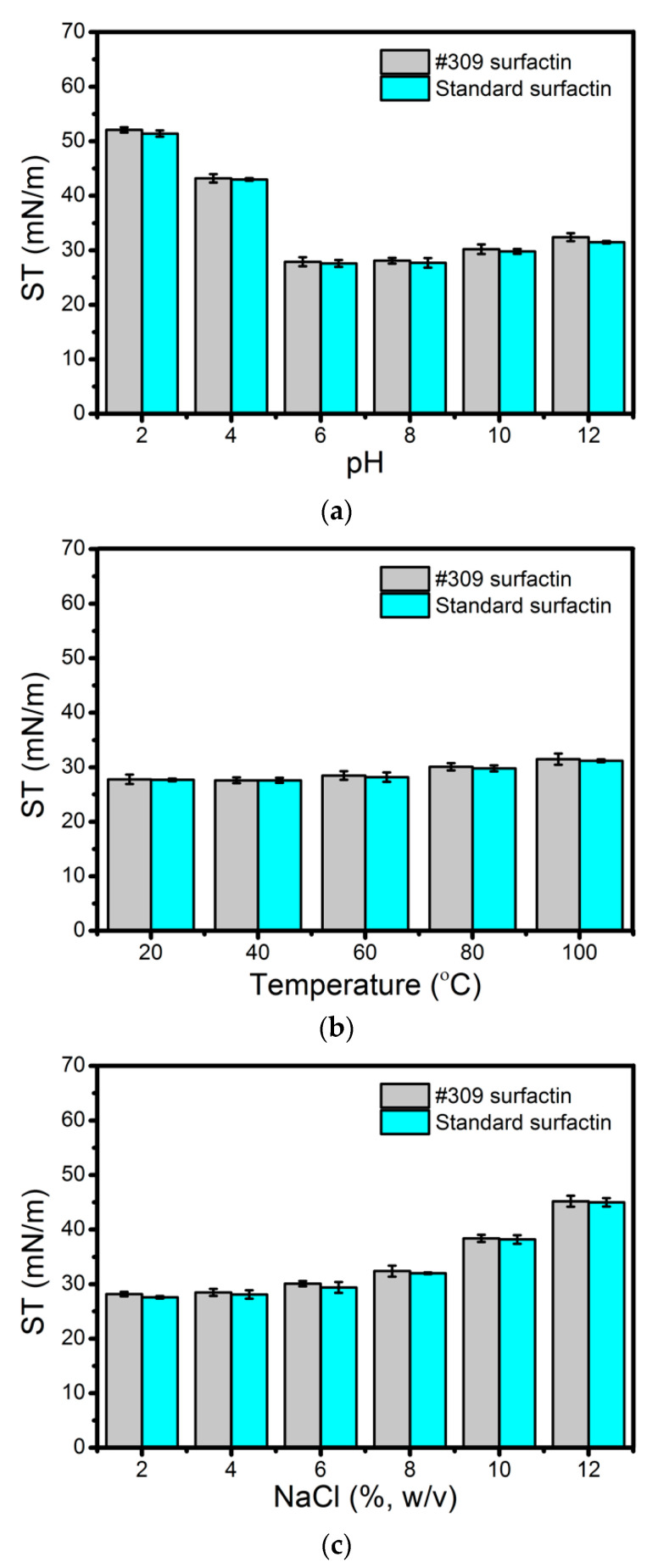
Stability studies using the biosurfactant produced by *Bacillus subtilis* #309 when grown in mineral salt medium (MSM) supplemented with soap-derived crude glycerol and surfactin standard (Merck, Darmstadt, Germany), under different pH values (**a**), temperatures (**b**) and salinities (**c**). Measurements were performed at room temperature (25 °C) after incubation at different pHs, temperatures and salinities for 2 h. The reference surface tension value was 70.4 mN/m. The results represent the mean ± standard deviation of three independent experiments performed in triplicate.

As can be seen in Figure 4a, the biosurfactant activity remained stable at pH values ranging from 6 to 12. However, at a low pH, some loss of activity could be observed. In our opinion, increasing the pH above 6.0 probably increased the negative charge of the polar head of surfactin, where the p*K*_a_ values of Asp and Glu are around 4.3 and 4.5, thus enhancing its solubility in water. The maximum surface activity of surfactin was observed at pHs 6.0–8.0, as the p*K*_a_ value of non-dissociated surfactin is about 6.0 [49]. Under highly acidic conditions (pH 2.0 and 4.0) surfactin precipitates, thus leading to much lower surface activity. The results produced by the surfactin secreted by *B. subtilis* #309 are similar to those obtained for the surfactin standard (Figure 4a). Similar results regarding the stability at different pH values of lipopeptide biosurfactants produced by *Bacillus* strains have been reported [47,50].

The biosurfactant produced by *B. subtilis* #309 was found to be stable after incubation for 2 h at temperatures ranging from 20 to 100 °C (Figure 4b). Moreover, it was also stable when exposed to NaCl concentrations up to 8% (*w/v*) (Figure 4c). Similar results were obtained for surfactin standard solutions (Figure 4a,b). Several studies on the biosurfactants’ stability at high temperatures and salinities have been reported [51,52,53]. The good stability of the analyzed biosurfactant at all tested temperatures and NaCl concentrations broadens the scope of its applicability in many industrial fields, from pharmaceuticals, food and detergents to enhanced oil recovery.

### 2.6. Fourier Transform Infrared Spectroscopy (FTIR) Studies

**Figure 5:**
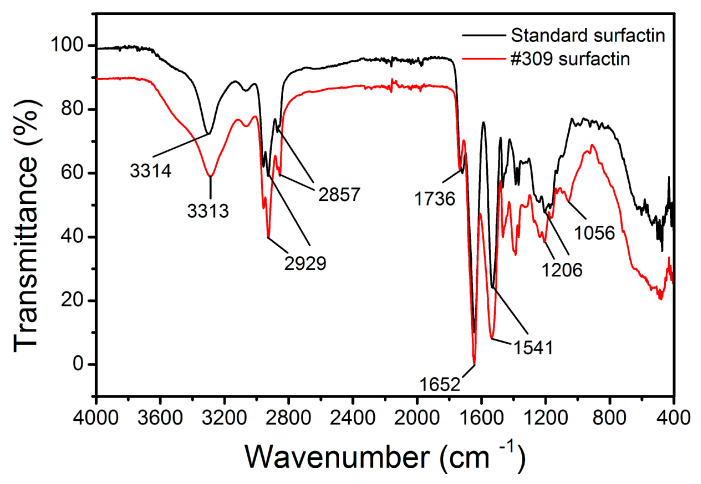
FTIR spectra of the surfactin produced by *Bacillus subtilis* #309 grown in mineral salt medium (MSM) supplemented with soap-derived crude glycerol and surfactin standard (Merck, Darmstadt, Germany).

The IR spectra obtained for the *B. subtilis* #309 biosurfactant and surfactin standard are shown in Figure 5. Both spectra showed the characteristic bands corresponding to the peptide component at 3300–3400 cm^−1^ (N-H stretching mode), 1650–1700 cm^−1^ (stretching mode of the CO–N bond) and at 1520–1550 cm^−1^ from the deformation mode of the N–H bond combined with the C–N stretching mode. On the other hand, the presence of an aliphatic chain indicated by the C–H modes at 2840–3000 cm^−1^ was also observed. The absorbance at 1620–1660 cm^−1^ belonged to the C=O stretching vibration of the amide I region [54], while a band observed at 1735–1750 cm^−1^ is due to a carbonyl group [55]. Compared with that for the surfactin standard, the absorbance intensity increased at 1380–1460 cm^−1^ for the *B. subtilis* #309 surfactin. This region corresponds to the –C–CH_2_ and –C–CH_3_ group vibrations in aliphatic chains and indicates that the *B. subtilis* #309 surfactin probably contained a few impurities of different chemical structures. However, the similarity between the two FTIR spectra confirmed that the biosurfactant produced by *B. subtilis* #309 had a similar structure and functional groups to the surfactin standard. The FTIR spectra are similar to others reported in the literature [21,56].

## 3. Material and Methods

### 3.1. Chemicals and Reagents

All chemicals and reagents were of analytical or LC-MS grade, purchased from Merck Co. (Darmstadt, Germany). Four different samples of crude glycerol from biodiesel (G1 and G2), soap (G3) and stearin (G4) production were obtained from Grupa Azoty, Orlen, and Lotos companies located in Poland. The composition of the four crude glycerol samples was previously reported [57], and is listed in Tables S1 and S2.

### 3.2. Microorganism

The previously reported bacterial strain *B. subtilis* #309, isolated from a crude oil sample from a Brazilian oil field [58], was used in all experiments. For long-term preservation, the strain was stored in 20% glycerol (*v/v*), at −80 °C, in the Department of Biotechnology and Food Microbiology, Wrocław University of Environmental and Life Sciences, Wrocław, Poland.

### 3.3. Culture Conditions

The modified MSM reported by Pereira et al. [21] was used for biosurfactant production by *B. subtilis* #309. The MSM consisted of (g/L): NH_4_NO_3_ 4.0, Na_2_HPO_4_ 5.0, KH_2_PO_4_ 2.0, and MgSO_4_ × 7H_2_O 0.2. Pure glycerol (POCH, Poland) or crude glycerol from different sources was added as a carbon source to the MSM at a concentration of 20 or 40 g/L, which corresponded to C/N ratios of 5.5 and 11, respectively. All culture media were adjusted to pH 7.0. Cultures were performed in 100 mL shake flasks containing 30 mL of MSM and incubated at 37 °C and 160 rpm in a rotary shaker (Innova 44, New Brunswick Scientific, Eppendorf AG, Hamburg, Germany) for 96 h. Each flask was inoculated with 1% of a pre-culture of *B. subtilis* #309 grown under the same conditions. Samples were taken regularly from the individual flasks and centrifuged at 4500× *g*. The biomass concentration was determined gravimetrically after drying the cells at 105 °C. The cell-free supernatants were used to measure the ST and glycerol consumption. The concentration of glycerol was determined by high-performance liquid chromatography (HPLC, UltiMate 3000, Dionex-Thermo Fisher Scientific, London, UK) equipped with a HyperRez Carbohydrate H^+^ Column (Thermo Fisher Scientific, London, UK) and a refractive index (RI) detector (Shodex, Ogimachi, Japan). Experiments were conducted in three independent experiments performed in triplicate.

### 3.4. Surfactin Quantification

LC-MS methods previously reported for surfactin quantification [59] were used with modifications. Briefly, culture samples were centrifuged (10,000× *g*, 15 min, 4 °C), and clear supernatants were diluted 10–100-fold with methanol, mixed and centrifuged again. 1 µL of each sample was injected onto an analytical LC column. Surfactin solutions for the calibration curve were prepared as follows: surfactin standard solution (1 mg/mL in methanol) was diluted 10-fold in methanol to a final concentration of 100 µg/mL. Next, a dilution series (in a concentration range between 100 and 1.5625 µg/mL) was prepared in methanol. 1 µL of each sample was injected onto an analytical LC column.

A Dionex UltiMate 3000 RSLC (Thermo Fisher Scientific, Waltham, MA, USA) UHPLC system, coupled with a MaXis Impact QTOF (Bruker, Bremen, Germany), was used for the LC-MS analysis of samples. A Kinetex XB-C18 (1.7 µm, 100 A, 2.1 × 100 mm) analytical column (Phenomenex, Torrance, CA, USA) with a SecurityGuard C18 guard column (Phenomenex, Torrance, CA, USA), kept at 30 °C, was used for the LC separation of surfactin. A 20 min LC gradient of water +0.1% formic acid (solvent A) and acetonitrile +0.1% formic acid (solvent B) was used for elution (0.3 mL/min): 0 min 60% B, 1 min 60% B, 2 min 90% B, 10 min 95% B, 10.1 min 98% B, 13 min 98% B, 13.5 min 60% B, 18 min 60% B. Samples were injected into the electrospray ionization (ESI) source and measured in the positive ionization mode, using the following settings: capillary voltage 3500 V, nebulizer 1.5 bar, dry gas 8 L/min, dry temp 180 °C. Data were collected for 400–1300 *m/z*. Next, the data obtained were processed with the Compass DataAnalysis software package (Bruker, Bremen, Germany). Internal mass calibration with a sodium formate solution was performed with a DataAnalysis v4.1, whereas extracting base peak chromatograms (900.0 ± 0.5–1200.0 ± 0.5 *m/z*) and smoothing chromatograms (Gauss, 0.1 sec, 3 cycles) were performed with QuantAnalysis v2.1. Then, the peak areas between 5.5 and 10 min were summed up and used for surfactin quantification. Spreadsheet software (Microsoft Excel; Microsoft, Redmond, WA, USA) was used to further analyze the obtained data, namely, to determine the calibration curves and surfactin concentration in samples. Means, standard deviations (SD) and relative standard deviations (RSD) were calculated. Each sample was injected at least twice on the LC column.

### 3.5. Surfactin Structure Identification

LC-MS/MS methods were used for the structural identification of surfactin analogs. Briefly, culture samples were prepared and analyzed with UHPLC-MS/MS, as described above. Samples were injected into the ESI source and measured in the positive ionization mode, using the following settings: capillary voltage 3500 V, nebulizer 1.5 bar, dry gas 8 L/min, dry temp 180 °C. Data were collected for 400–1300 *m/z,* and selected ions in the range of 800–1300 *m/z* were fragmented using CID mode and collision energy between 45 and 55 eV. Precursor ions were selected with a tolerance of 0.2 *m/z*. Raw data were analyzed using DataAnalysis v4.1 software (Bruker, Bremen, Germany).

### 3.6. Emulsification Index (E_24_) and Surface-Activity Determination

The emulsification activity was tested by adding 2 mL of culture supernatant to 2 mL of n-hexadecane in a glass test tube. The mixture was then vortexed at high speed for 2 min. The stability of the emulsion was determined after 24 h. All the measurements were performed in triplicate. The emulsification index value (E_24_, %) was calculated according to the following equation:

(2) $$E_{24}\left(\%\right)=\frac{\mathrm{height}\;\mathrm{of}\;\mathrm{the}\;\mathrm{emulsion}\;\mathrm{layer}}{\mathrm{total}\;\mathrm{height}\;\mathrm{of}\;\mathrm{the}\;\mathrm{mixtures}}\times 100$$

Measurements of the ST of the cell-free supernatants were carried out using a Krüss K6 Tensiometer (KRÜSS GmbH, Berlin, Germany) according to the Du Noüy ring method as described elsewhere [10]. For these ST measurements, the produced biosurfactants of all cultures were diluted 10-fold (ST^−1^) and 100-fold (ST^−2^) with distilled water, respectively. All the measurements were performed at room temperature (25 °C) and are reported as the mean ± standard deviation (*n* = 9).

### 3.7. Surfactin Recovery

In brief, surfactin was isolated and purified by acid precipitation [60], namely using 6 M HCl until the pH = 2.0, and kept at 4 °C overnight. Afterward, the crude biosurfactant was pelleted by centrifugation at 9500× *g* for 20 min at 4 °C. Subsequently, the partially purified pellet was dissolved in deionized water, and the pH was adjusted to 7.0 using concentrated NaOH. For additional purification, the biosurfactant was separated using solid-phase extraction (SPE). Crude biosurfactant was loaded onto Chromabond C_18_ SPE cartridges and further washed with 40, 60, 80 and 100% acetonitrile–water (*v/v*). The 80% acetonitrile–water (*v/v*) elution (containing surfactin) was concentrated with nitrogen drying and further analyzed using Fourier transform infrared spectroscopy (FTIR). The surfactin was detected by spraying 0.1% bromothymol blue in 10% ethanol on the TLC plate. The presence of the biosurfactant was detected by the formation of brown spots. The retention time (Rf) of the purified surfactin was compared with a standard surfactin (Merck, purity > 98%).

### 3.8. Critical Micelle Concentration (CMC) of the Purified Surfactin

The CMC was determined by measuring the ST at various dilutions of the purified surfactin solution (250 mg/L) and compared with the surfactin standard (purchased from Merck (Darmstadt, Germany). The surfactin solutions were prepared and diluted in a 10 mM HEPES buffer (pH 7.4). The ST was plotted versus surfactin concentration to determine the CMC. All the measurements were performed in three independent experiments and analyzed in triplicate.

### 3.9. Surfactin Stability

Surfactin stability was evaluated in a wide range of pH, temperatures and salinities, according to a previous report [61]. For the pH stability analysis, the biosurfactant solution (50 mg/L) was incubated at different pHs (2, 4, 6, 8, 10, 12) at 20 °C. In turn, to investigate the temperature stability of the biosurfactant, its solution was incubated at different temperatures (20, 40, 60, 80 and 100 °C) at pH 6.0. Furthermore, the effect of the different salinity on surfactin activity was investigated in the range of sodium chloride concentrations from 2 to 12% (*w/v*) at pH 6.0 at 20 °C. All samples were kept at the defined temperature, pH and salinities for 2 h. The ST of each sample was determined as described above, and all measurements were performed in three independent experiments performed in triplicate.

### 3.10. Fourier Transform Infrared Spectroscopy (FTIR)

The purified surfactin was characterized by FTIR spectroscopy and compared with a surfactin standard purchased from Merck (Darmstadt, Germany). For FTIR spectroscopic analysis, the sample was prepared by grinding 1 mg of dried surfactin with 100 mg of KBr and pressed at 400 MPa to acquire a transparent pellet. The IR spectra were recorded using the Thermo Scientific Nicolet iS50 FTIR spectrometer (Thermo Fisher Scientific, Waltham, MA, USA) at room temperature (25 °C). The main functional groups of surfactin were observed between 400 and 4000 wavenumbers (cm^−1^) at a resolution of 2 cm^−1^.

## 4. Conclusions

*B. subtilis* #309 was able to grow in a mineral medium containing waste glycerol derived from different industrial processes as a carbon source. The highest biosurfactant production (2.8 g/L) was achieved by cultivating this strain in a medium containing waste glycerol derived from soap production. The biosurfactants produced using the different crude glycerols were composed of six surfactin analogs (mainly C_13_, C_14_ and C_15_ surfactin). The biosurfactant showed stability under various extreme conditions, exhibiting optimum activity at pH 6.0–8.0, 20–40 °C and 2–4% NaCl (*w/v*). This study provides the first evidence that surfactin can be produced using a culture medium containing stearin-derived and soap-derived crude glycerol. To reduce the costs of surfactant production, soap-derived glycerol was used as a promising cheap and renewable carbon source. Application of this raw material allowed for a five-fold increase in surfactant biosynthesis when compared to the same medium with pure glycerol as substrate. Despite the promising results, the scalability of the process should be further investigated with a view to the development of a sustainable bioprocess.

## Figures and Tables

**Table 1:** Surface tension values [ST^−1^ and ST^−2^ (mN/m)] obtained for the cell-free supernatants of cultures of *Bacillus subtilis* #309 grown in MSM supplemented with 20 and 40 g/L of different waste glycerol samples and pure glycerol. The culture broth supernatants were diluted 10 times (ST^−1^) or 100 times (ST^−2^) with distilled water, and the surface tension was measured as described in Materials and Methods. The cultures were performed at 37 °C and 160 rpm for 96 h. The results represent the mean ± standard deviation of three independent experiments performed in triplicate.

Test Parameters	Time (h)	G1 (Biodiesel)	G2 (Biodiesel)	G3 (Stearin)	G4 (Soap)	G5 (Pure Glycerol)
**20 g/L glycerol**						
ST^−1^ (mN/m)	0	69.7 ± 0.11	69.4 ± 0.07	69.1 ± 0.31	69.7 ± 0.03	70.1 ± 0.07
	24	29.4 ± 0.13	29.7 ± 0.14	29.9 ± 0.25	29.1 ± 0.04	29.4 ± 0.06
	48	29.9 ± 0.22	29.7 ± 0.14	63.4 ± 0.17	28.9 ± 0.23	63.1 ± 0.07
	72	42.7 ± 0.15	44.1 ± 0.08	67.8 ± 0.32	28.7 ± 0.16	67.7 ± 0.22
	96	67.2 ± 0.12	64.5 ± 0.11	68.4 ± 0.27	28.6 ± 0.17	69.4 ± 0.26
ST^−2^ (mN/m)	0	70.2 ± 0.09	70.2 ± 0.21	69.7 ± 0.03	70.1 ± 0.25	70.3 ± 0.31
	24	49.8 ± 0.12	47.2 ± 0.18	49.2 ± 0.19	51.4 ± 0.27	52.4 ± 0.36
	48	68.4 ± 0.17	68.6 ± 0.11	43.8 ± 0.24	41.2 ± 0.16	68.8 ± 0.18
	72	68.9 ± 0.03	69.8 ± 0.03	43.1 ± 0.21	38.7 ± 0.21	69.7 ± 0.05
	96	69.8 ± 0.13	70.1 ± 0.11	48.4 ± 0.26	39.1 ± 0.31	70.2 ± 0.31
**40 g/L glycerol**						
ST^−1^ (mN/m)	0	69.3 ± 0.21	69.1 ± 0.07	68.5 ± 0.04	70.0 ± 0.16	70.2 ± 0.06
	24	29.2 ± 0.28	31.2 ± 0.28	32.0 ± 0.09	32.3 ± 0.06	34.2 ± 0.24
	48	28.9 ± 0.19	30.2 ± 0.15	31.4 ± 0.30	28.6 ± 0.02	31.5 ± 0.14
	72	29.0 ± 0.15	30.5 ± 0.16	31.6 ± 0.24	28.6 ± 0.03	31.8 ± 0.13
	96	29.1 ± 0.03	30.7 ± 0.08	31.6 ± 0.12	28.5 ± 0.05	31.9 ± 0.12
ST^−2^ (mN/m)	0	70.1 ± 0.12	69.8 ± 0.06	69.8 ± 0.18	70.1 ± 0.11	70.2 ± 0.03
	24	43.0 ± 0.23	43.9 ± 0.05	43.4 ± 0.04	48.2 ± 0.05	51.8 ± 0.22
	48	41.5 ± 0.04	42.3 ± 0.05	43.8 ± 0.22	40.8 ± 0.09	43.7 ± 0.02
	72	42.2 ± 0.07	42.1 ± 0.08	43.1 ± 0.10	38.2 ± 0.12	45.7 ± 0.17
	96	42.5 ± 0.06	42.9 ± 0.12	48.4 ± 0.06	36.4 ± 0.19	49.2 ± 0.07

**Table 2:** Relative abundance (%) of surfactin structural analogs present in surfactin standard (Merck) and in the cultures of *Bacillus subtilis* #309 grown in mineral salt medium (MSM) supplemented with glycerol from different sources (G1, G2, G3, G4 or G5). Analogs with a relative abundance >1% were quantified. The results are presented as mean ± standard deviation. MS/MS spectra of surfactin variants, together with the elucidation of their structures, are shown in Supplementary Materials. Rt: retention time. ND: no data.

[M + H]^+^ (*m/z*)	Rt (min)	Surfactin Analog	Surfactin Standard	G1 (Biodiesel)	G2 (Biodiesel)	G3 (Stearin)	G4 (Soap)	G5 (Pure Glycerol)
994.64 ± 0.10	6.45	C_12_ Surfactin A	2.9 ± 0.1	1.1 ± 0.0	1.1 ± 0.0	2.0 ± 0.1	1.5 ± 0.0	<1.0
	7.00	C_13_ Surfactin B	1.0 ± 0.0	2.4 ± 0.1	2.2 ± 0.0	2.7 ± 0.1	2.5 ± 0.0	1.9 ± 0.0
	7.50	C_14_ Surfactin	<1.0	<1.0	<1.0	1.0 ± 0.0	<1.0	<1.0
1008.66 ± 0.10	6.82	C_13_ Surfactin A	12.8 ± 0.5	7.5 ± 0.1	8.0 ± 0.1	6.7 ± 0.1	10.2 ±0.1	6.5 ± 0.1
	6.94	C_13_ Surfactin A	<1.0	<1.0	<1.0	3.1 ± 0.1	3.1 ± 0.1	<1.0
	7.20	C_14_ Surfactin B	<1.0	2.1 ± 0.0	1.9 ± 0.0	2.0 ± 0.1	1.6 ± 0.0	1.7 ± 0.1
	7.50	C_14_ Surfactin B	<1.0	10.7 ± 0.1	8.9 ± 0.1	7.9 ± 0.2	5.3 ± 0.0	8.3 ± 0.1
	7.62	C_14_ Surfactin B	1.8 ± 0.0	<1.0	<1.0	2.4 ± 0.0	1.4 ± 0.1	<1.0
1022.68 ± 0.10	7.31	C_14_ Surfactin A	8.7 ± 0.2	34.0 ± 0.2	34.9 ± 0.1	28.4 ± 0.3	23.1 ± 0.3	34.6 ± 0.2
	7.42	C_14_ Surfactin A	23.8 ± 0.1	3.3 ± 0.1	3.2 ± 0.2	10.6 ± 0.2	8.8 ± 0.1	3.5 ± 0.2
	7.55	ND	<1.0	1.3 ± 0.1	1.2 ± 0.1	<1.0	<1.0	1.0 ± 0.1
	7.88	C_14_ Surfactin A	<1.0	3.0 ± 0.1	2.7 ± 0.0	2.0 ± 0.1	1.4 ± 0.0	2.0 ± 0.0
	8.07	C_15_ Surfactin B	3.6 ± 0.1	5.3 ± 0.0	4.9 ± 0.1	5.0 ± 0.1	5.7 ± 0.1	5.2 ± 0.1
1036.69 ± 0.10	7.83	C_15_ Surfactin A	36.9 ± 0.4	19.9 ± 0.1	22.0 ± 0.2	18.8 ± 0.2	27.3 ± 0.2	24.8 ± 0.2
	8.12	ND	1.0 ± 0.1	<1.0	<1.0	1.7 ± 0.1	1.8 ± 0.2	<1.0
	8.51	C_15_ Surfactin A	1.3 ± 0.0	1.7 ± 0.0	1.7 ± 0.1	1.4 ± 0.0	1.7 ± 0.1	1.5 ± 0.1
	8.82	C_17_ Surfactin B	<1.0	1.2 ± 0.0	1.0 ± 0.1	<1.0	<1.0	1.1 ± 0.1
1050.71 ± 0.10	8.50	C_16_ Surfactin A	1.0 ± 0.0	3.1 ± 0.1	3.3 ± 0.0	2.1 ± 0.1	2.2 ± 0.0	4.0 ± 0.1
	8.65	C_16_ Surfactin A	2.0 ± 0.0	<1.0	<1.0	<1.0	<1.0	<1.0

## Data Availability

The data presented in this study are available on request from the corresponding author.

## Supplementary Materials

The following are available online, Figure S1: Chromatograms of standard surfactin and *B. subtilis* #309 cell free supernatant, Figures S2–S6: MS/MS spectra of surfactin homologues, Figure S7: TLC analysis of surfactin, Tables S1–S2: Elemental composition of crude glycerol from different sources, Table S3: Relative abundance (%) of surfactin structural analogues.

## Sample Availability

Not available.
