# Supplementary material for: Sustainable Surfactin Production by Bacillus subtilis Using Crude Glycerol from Different Wastes
Source: Molecules. 2021 Jun 8;26(12):3488. doi: 10.3390/molecules26123488 (PMC8230125; doi:10.3390/molecules26123488)
Supplement: Supplementary file 1 [file molecules-26-03488-s001.zip › molecules-1221179-supplementary.pdf]

Supplementary materials

Sustainable surfactin production by *Bacillus subtilis* using crude glycerol from different wastes

Tomasz Janek [1,*], Eduardo J. Gudiña [2], Xymena Połomska [1], Piotr Biniarz [1,3], Dominika Jama [1], Lígia R. Rodrigues [2], Waldemar Rymowicz [1], Zbigniew Lazar [1]

[1] Department of Biotechnology and Food Microbiology, Wrocław University of Environmental and Life Sciences, 51-630, Wrocław, Poland

[2] Centre of Biological Engineering, University of Minho, 4710-057 Braga, Portugal

[3] Łukasiewicz Research Network – PORT Polish Center for Technology Development, 54-066 Wrocław, Poland

* Correspondence: tomasz.janek@upwr.edu.pl (TJ); Tel.: +48-71-320-7734

(a)

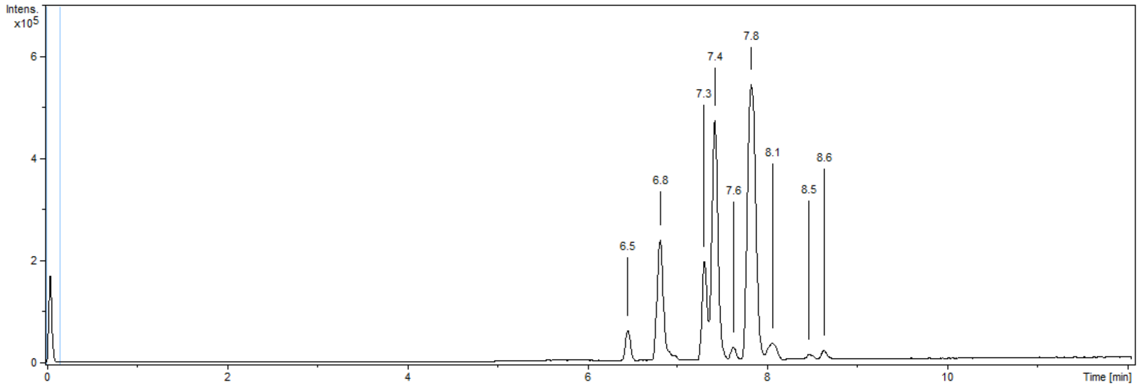

(b)

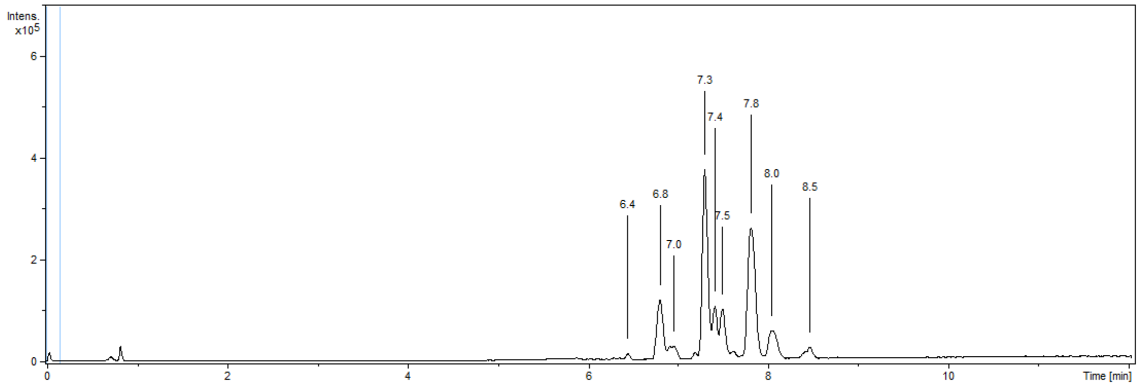

Figure S1. Chromatograms of standard surfactin (50 µg/mL) (a) and *Bacillus subtilis* #309 cell-free supernatant diluted 100 times with methanol (b). Retention times of selected surfactin peaks are marked. Data collected for m/z 900.0 ± 0.5 – 1200.0 ± 0.5 are shown.

MS/MS spectrum of the $[M+H]^+$ 994.64 m/z ion at Rt=6.50 min.

C12 surfactin A:

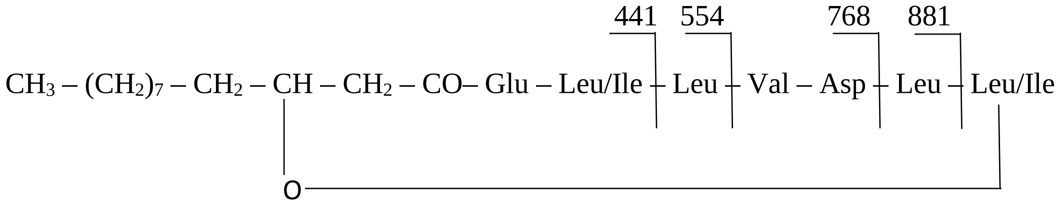

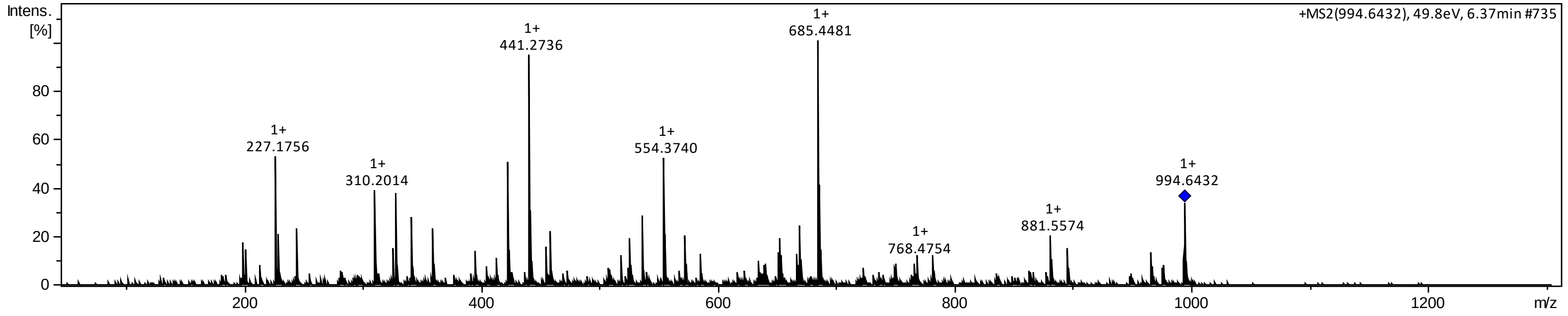

MS/MS spectrum of the $[M+H]^+$ 994.64 m/z ion at Rt=7.00 min.

C13 surfactin B:

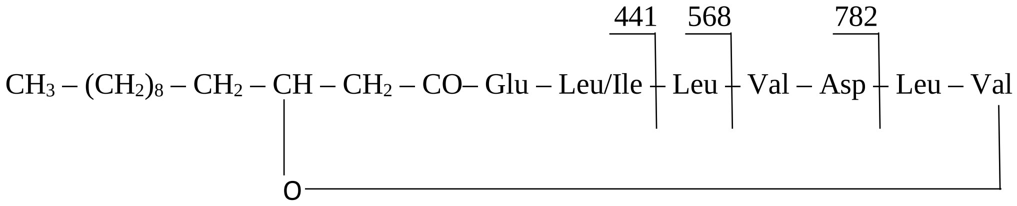

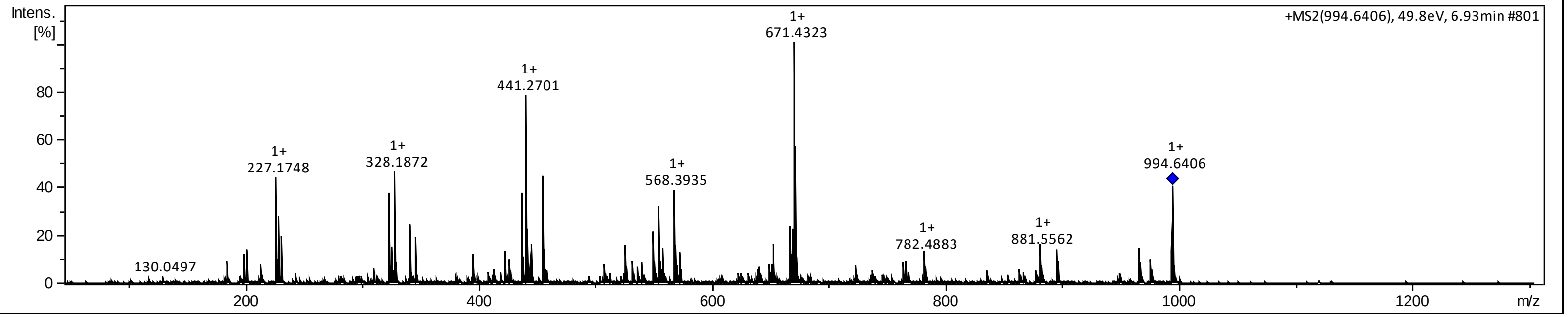

MS/MS spectrum of the $[M+H]^+$ 994.64 m/z ion at Rt=7.50 min.
C14 surfactin isoform:

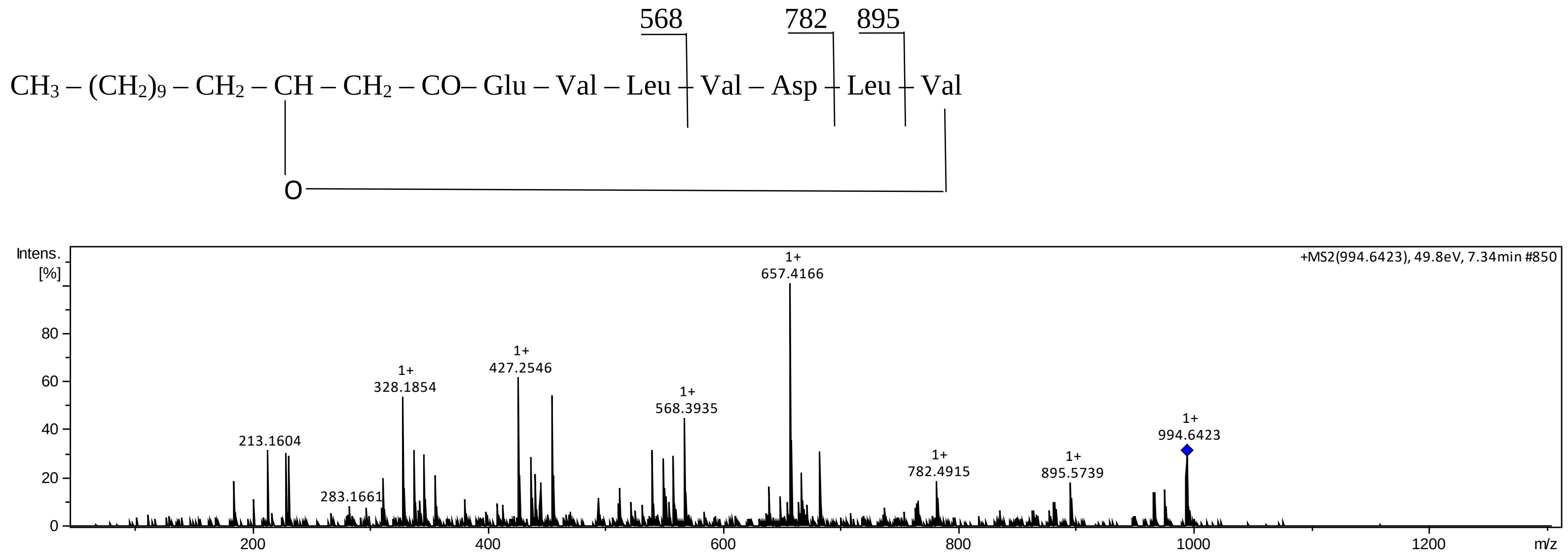

Figure S2. MS/MS spectra of [M+H]+ 994.64 m/z ions detected in culture supernatants of *B. subtilis* #309.

MS/MS spectrum of the $[M+H]^+$ 1008.66 m/z ion at Rt=6.82 min.

C13 surfactin A:

568 | 895

$CH_3 – (CH_2)_8 – CH_2 – CH – CH_2 – CO$– Glu – Leu/Ile – Leu | Val – Asp – Leu | Leu/Ile

O (lactone bond from CH to Leu/Ile)

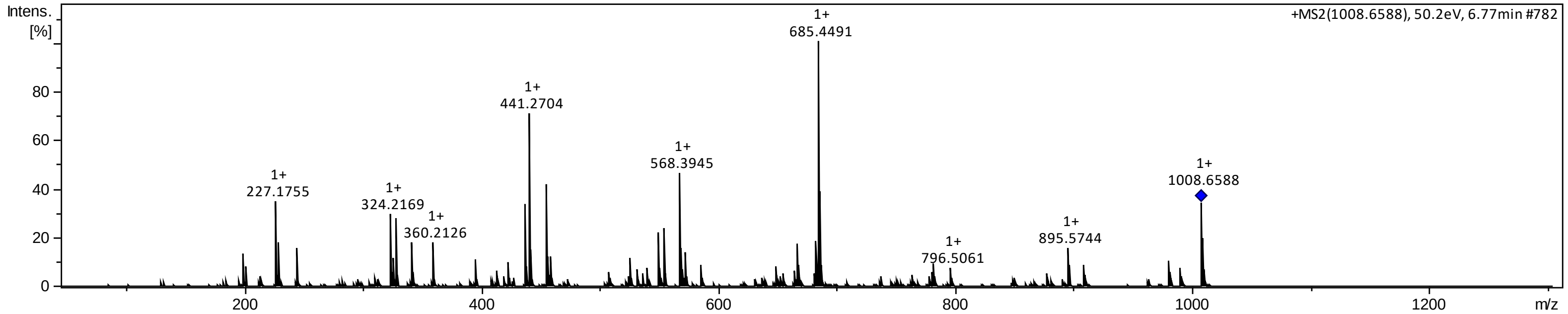

MS/MS spectrum of the $[M+H]^+$ 1008.66 m/z ion at Rt=6.94 min.

C13 surfactin A:

568 | 782 | 895

$CH_3 – (CH_2)_8 – CH_2 – CH – CH_2 – CO$– Glu – Leu/Ile – Leu | Val – Asp | Leu | Leu/Ile

O (lactone bond from CH to Leu/Ile)

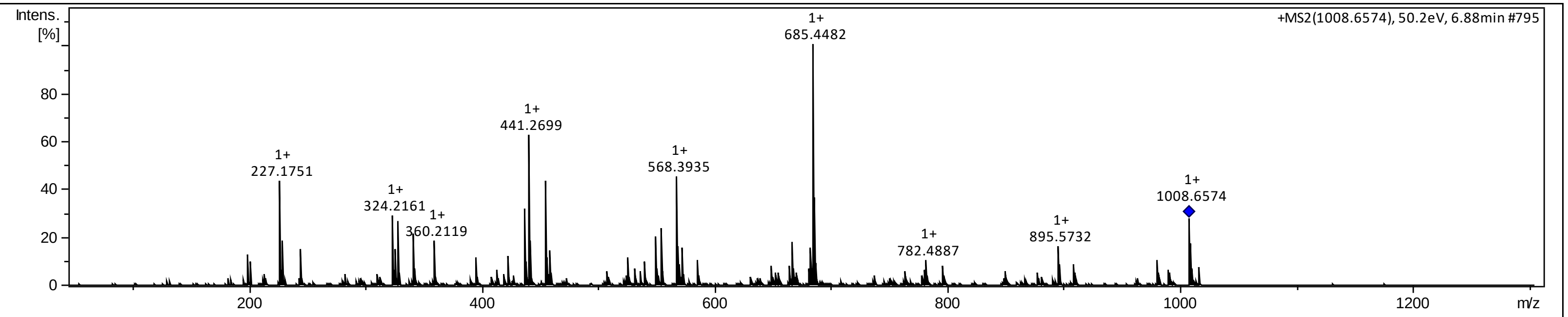

MS/MS spectrum of the $[M+H]^+$ 1008.66 m/z ion at Rt=7.20 min.

C14 surfactin B:

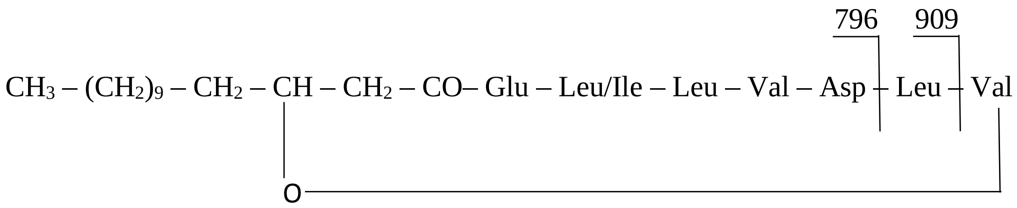

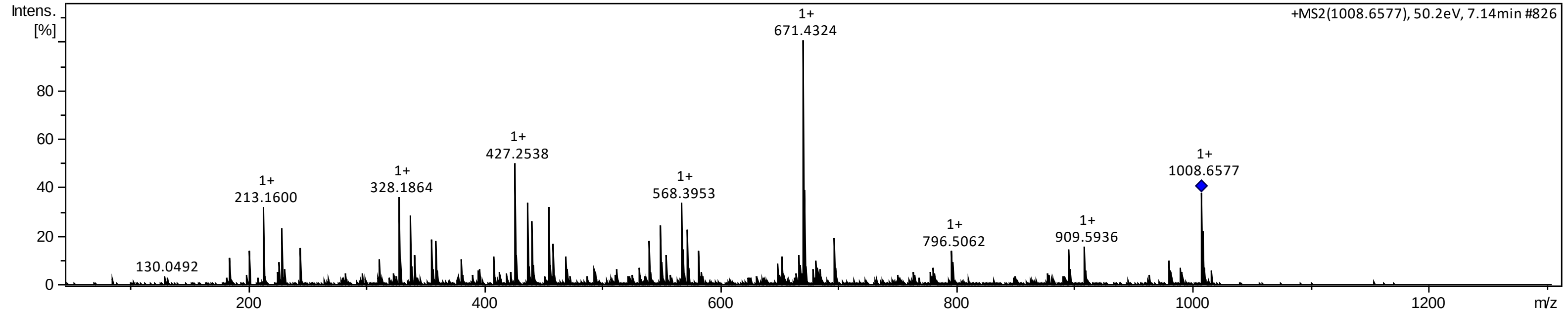

MS/MS spectrum of the $[M+H]^+$ 1008.66 m/z ion at Rt=7.50 min.

C14 surfactin B:

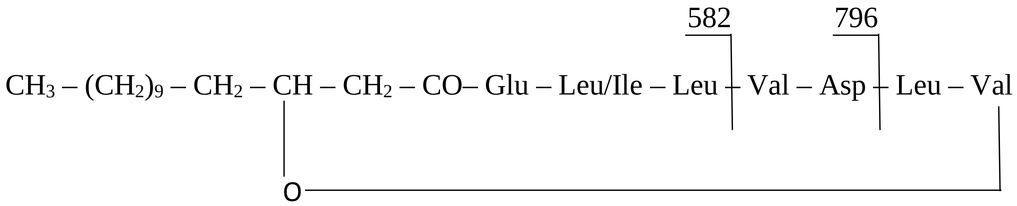

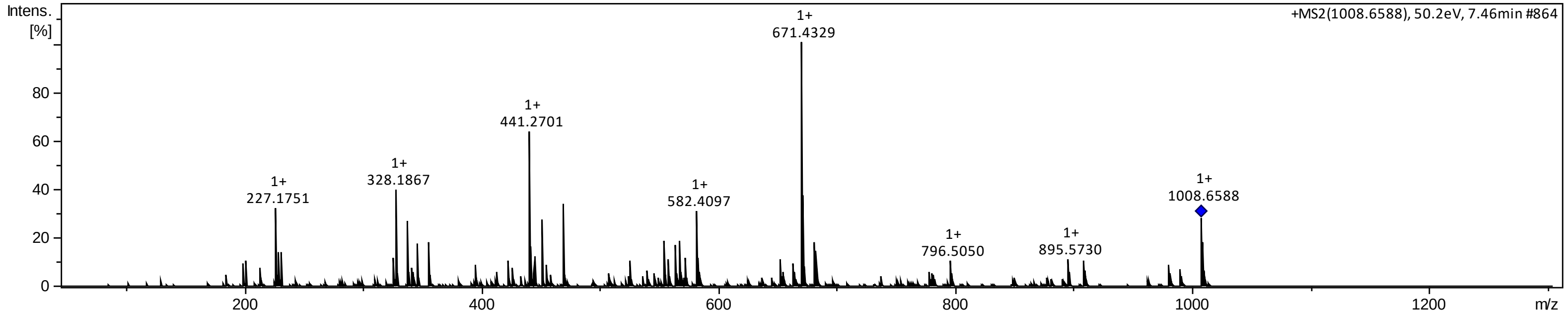

MS/MS spectrum of the $[M+H]^+$ 1008.66 m/z ion at Rt=7.62 min.

C14 surfactin B:

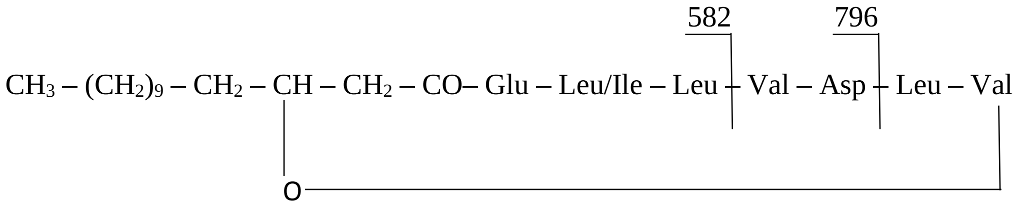

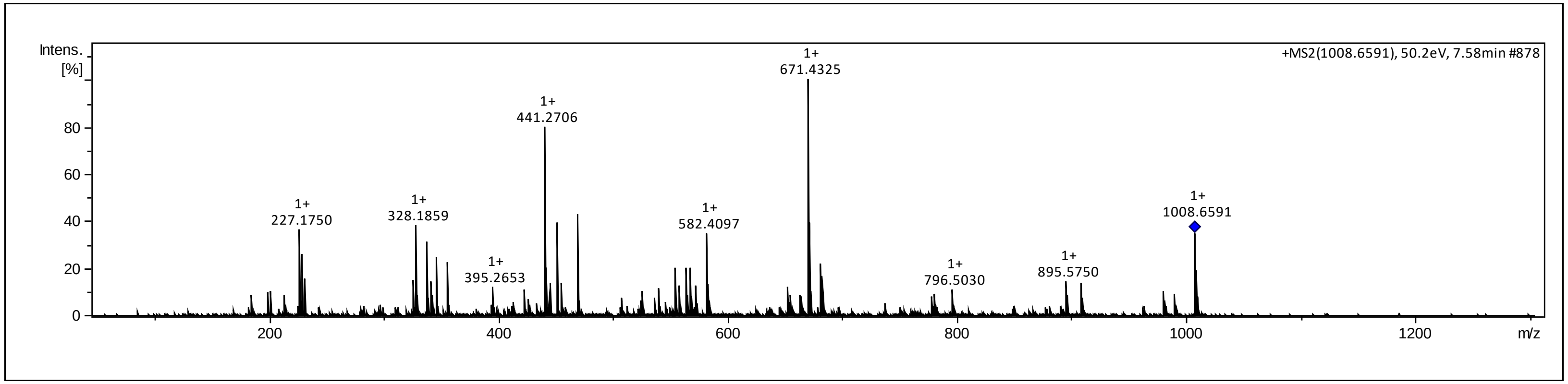

Figure S3. MS/MS spectra of [M+H]+ 1008.66 m/z ions detected in culture supernatants of *B. subtilis* #309.

MS/MS spectrum of the $[M+H]^+$ 1022.68 m/z ion at Rt=7.31 min.

C14 surfactin A:

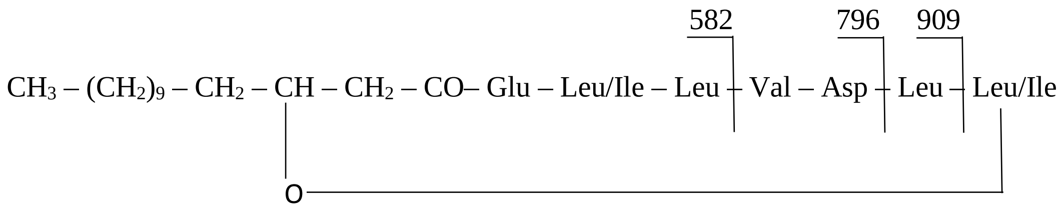

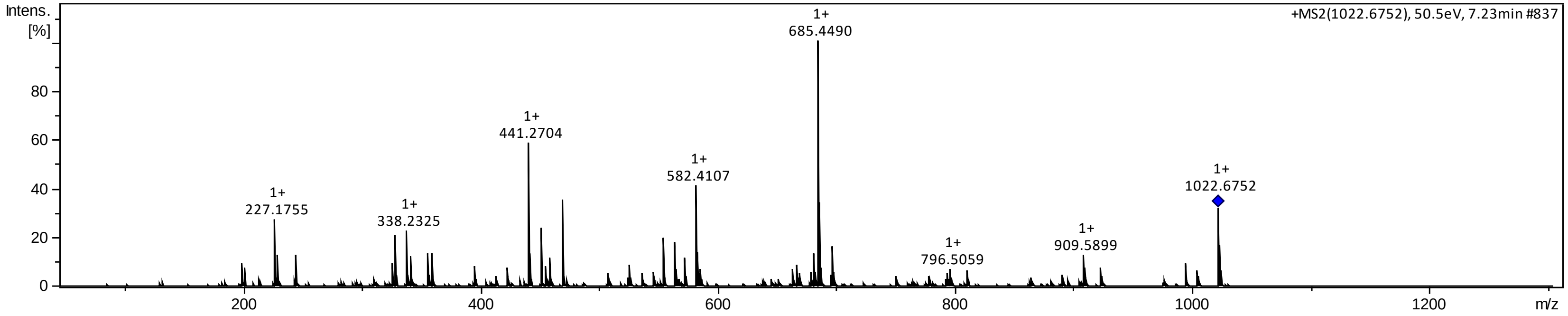

MS/MS spectrum of the $[M+H]^+$ 1022.68 m/z ion at Rt=7.38 min.

C14 surfactin A:

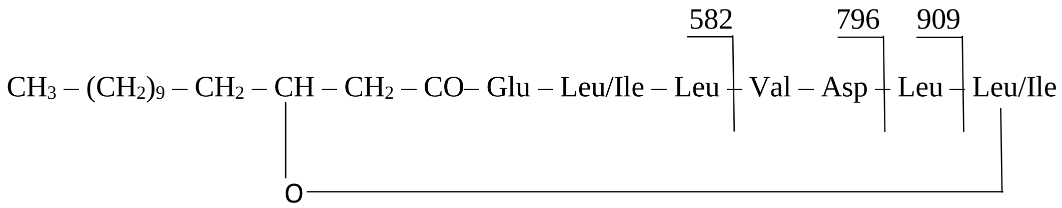

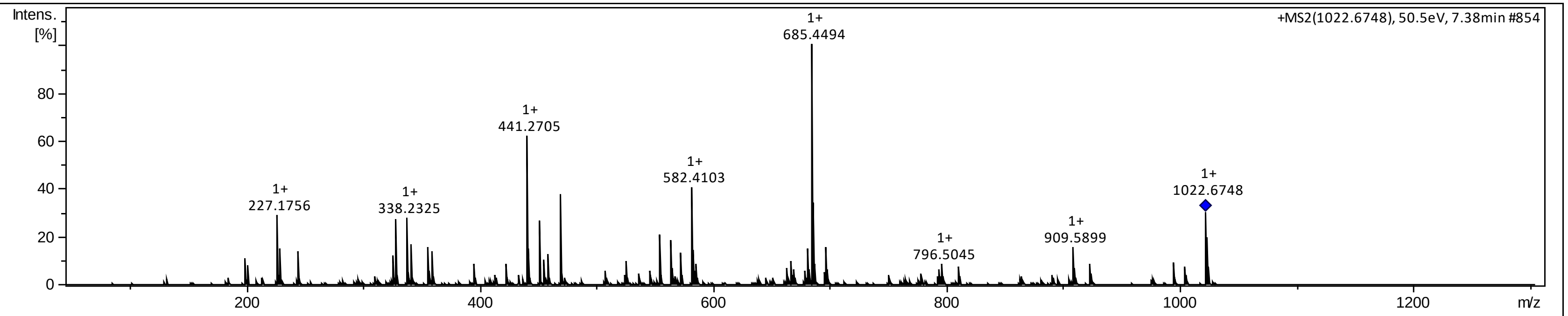

MS/MS spectrum of the $[M+H]^+$ 1022.68 m/z ion at Rt=7.55 min.
Good quality spectrum could not be collected due to the high intensity of coeluting ions $[M+H]^+$ 1008.66 m/z and 1036.69 m/z

MS/MS spectrum of the $[M+H]^+$ 1022.68 m/z ion at Rt=7.88 min.
C14 surfactin A:

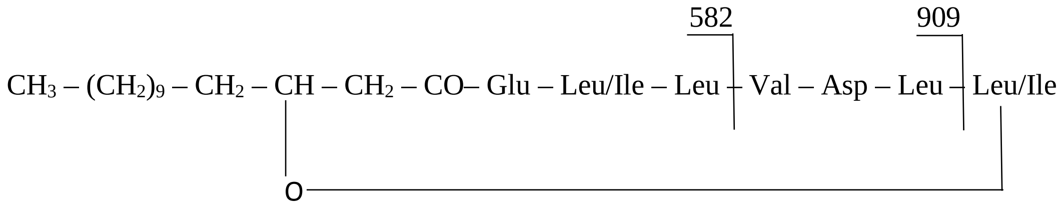

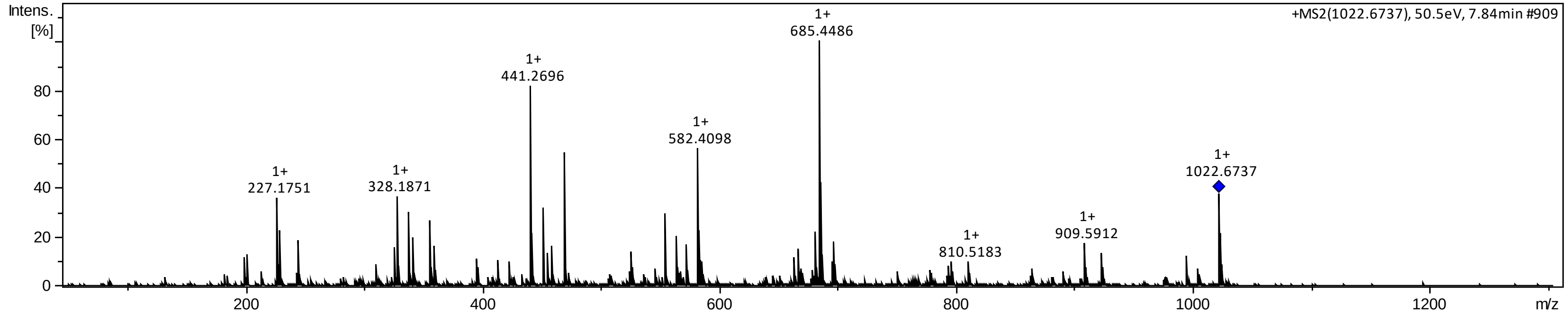

MS/MS spectrum of the $[M+H]^+$ 1022.68 m/z ion at Rt=8.07 min.
C15 surfactin B:

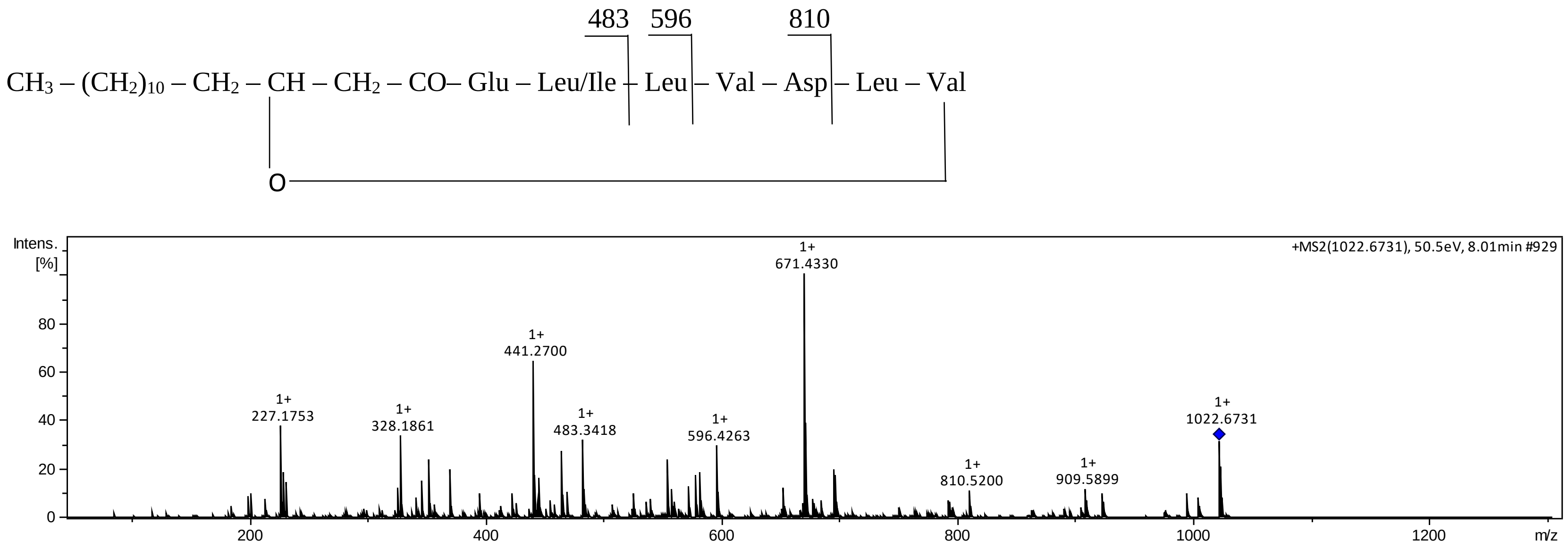

Figure S4. MS/MS spectra of [M+H]+ 1022.68 m/z ions detected in culture supernatants of *B. subtilis* #309.

MS/MS spectrum of the $[M+H]^+$ 1036.69 m/z ion at Rt=7.83 min.
C15 surfactin A:

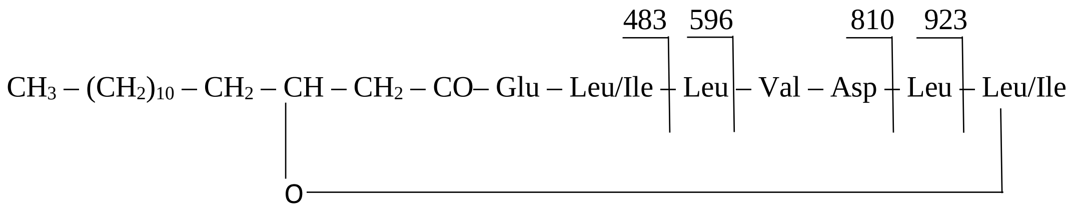

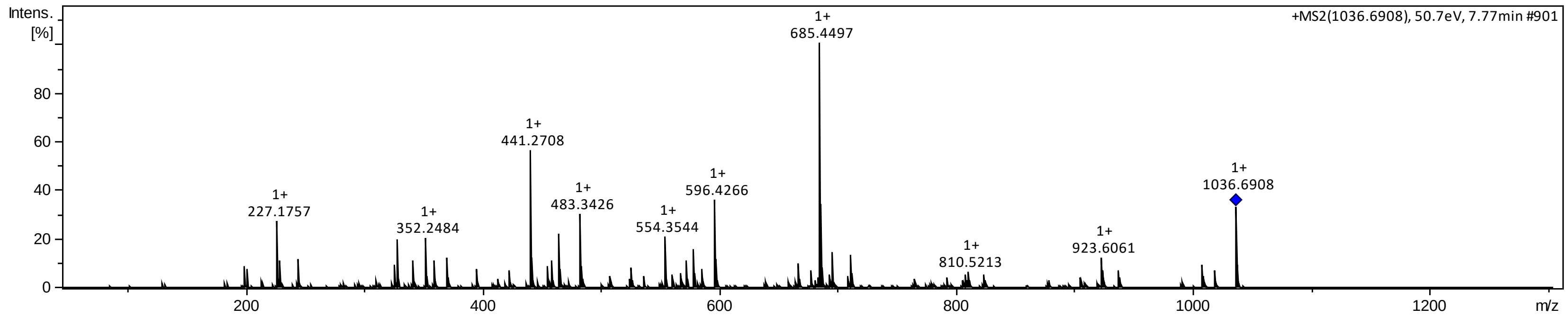

MS/MS spectrum of the $[M+H]^+$ 1036.69 m/z ion at Rt=8.12 min.
Good quality spectrum could not be collected due to the high intensity of coeluting ions $[M+H]^+$ 1022.68 m/z

MS/MS spectrum of the $[M+H]^+$ 1036.69 m/z ion at Rt=8.51 min.
C15 surfactin A:

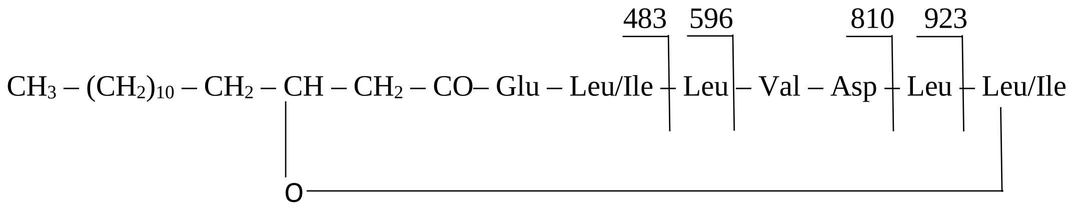

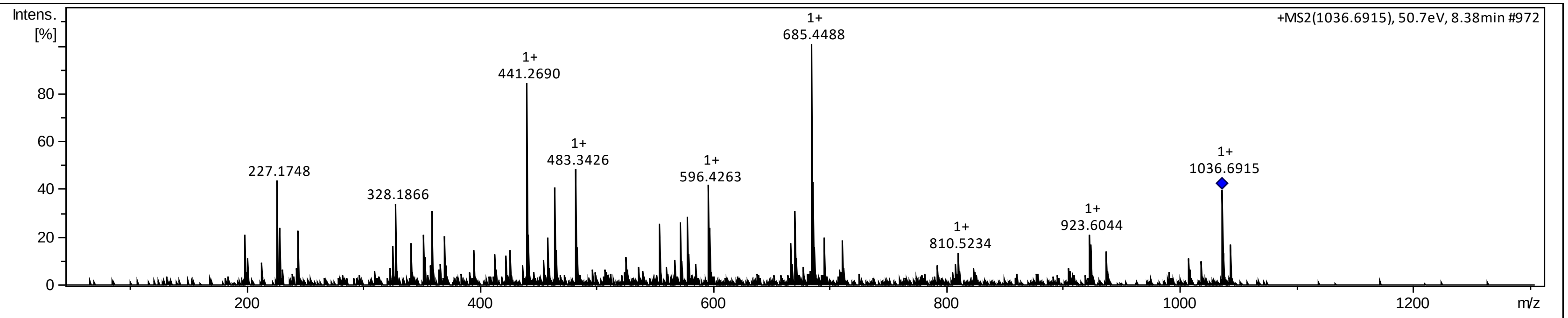

MS/MS spectrum of the $[M+H]^+$ 1036.69 m/z ion at Rt=8.82 min.
C17 surfactin B:

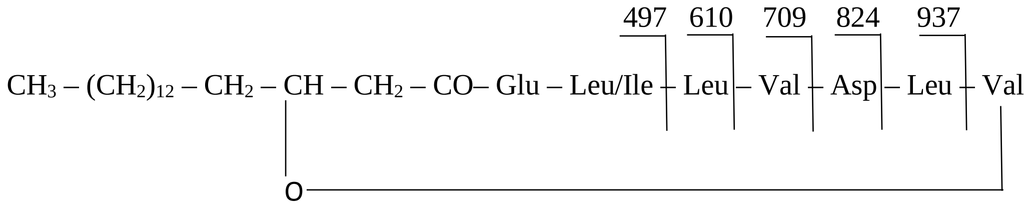

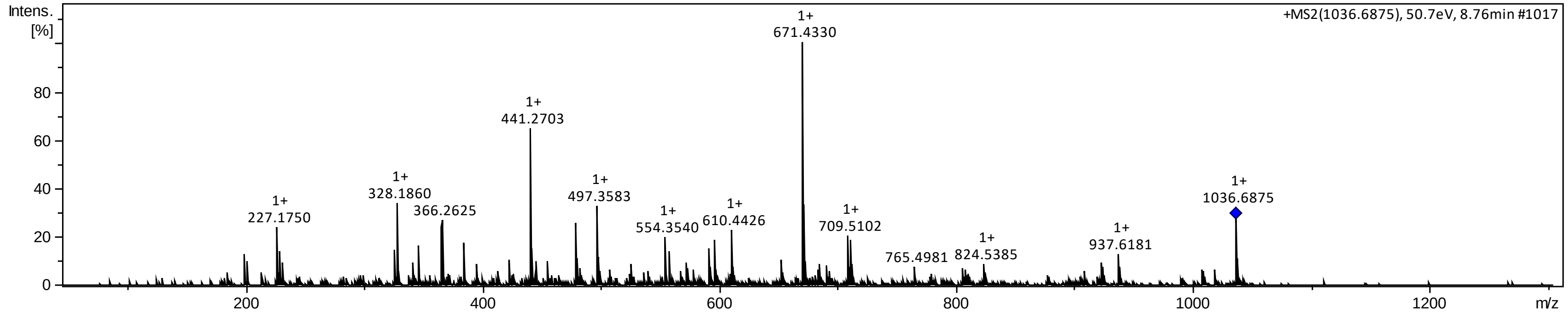

Figure S5. MS/MS spectra of [M+H]+ 1036.69 m/z ions detected in culture supernatants of *B. subtilis* #309.

MS/MS spectrum of the $[M+H]^+$ 1050.71 m/z ion at Rt=8.50 min.
C16 surfactin A:

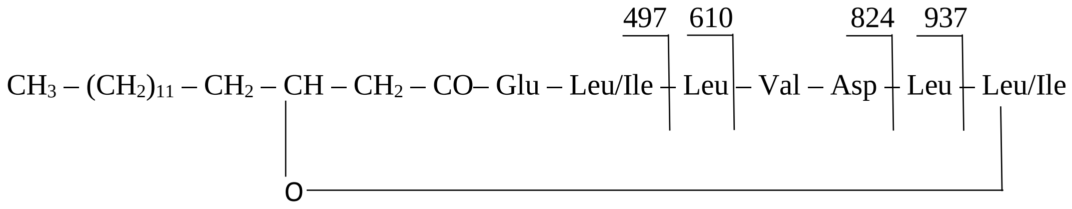

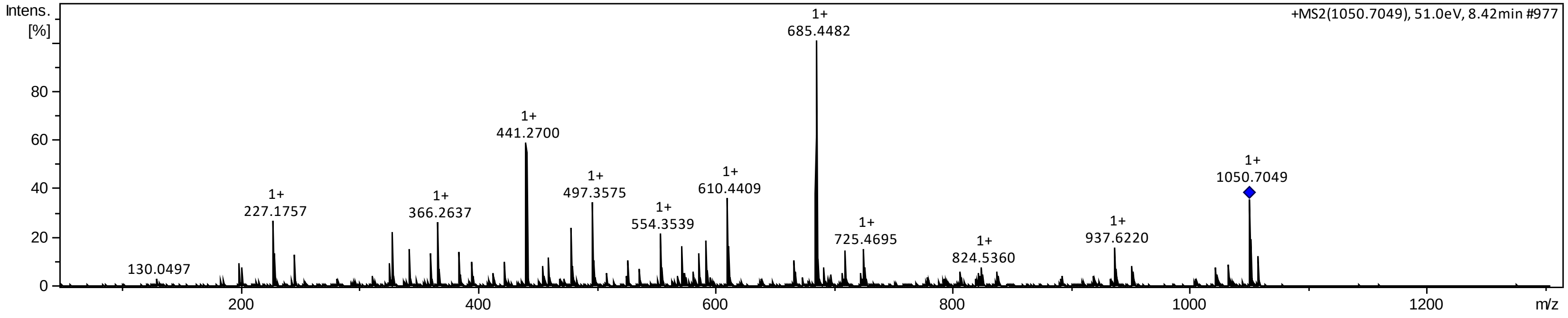

MS/MS spectrum of the $[M+H]^+$ 1050.71 m/z ion at Rt=8.65 min.
C16 surfactin A:

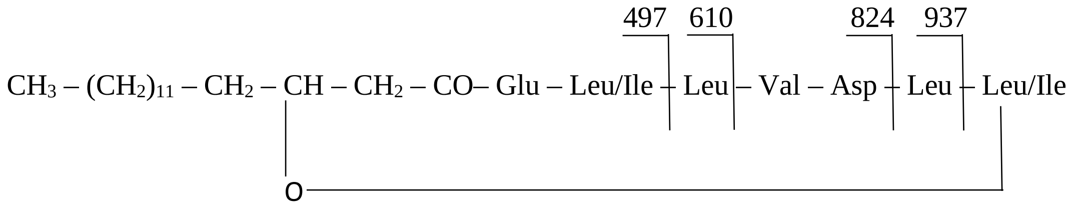

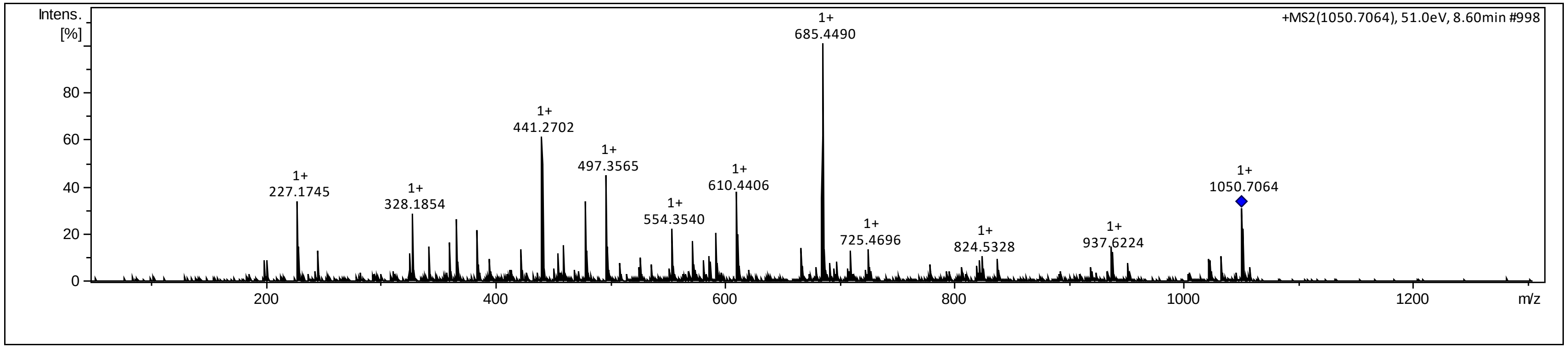

Figure S6. MS/MS spectra of [M+H]+ 1050.71 m/z ions detected in culture supernatants of *B. subtilis* #309.

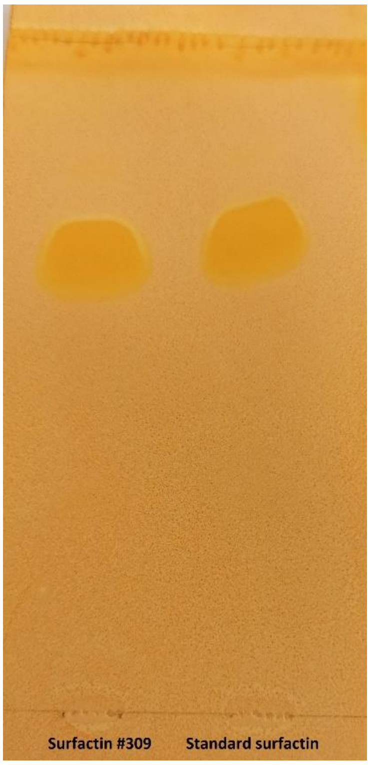

Figure S7. Thin-layer chromatography (TLC) analysis of lipopeptide produced by *B. subtilis* #309. Developed with chloroform/methanol/water (65:25:4 vol/vol/vol); detected with bromothymol blue.

Table S1. Composition of crude glycerol from different sources [1].

Symbol	G1	G2	G3	G4	G5
Waste product derived from [%]:	Biodiesel production	Biodiesel production	Stearin production	Soap production	Pure glycerol
Glycerol content	80	50	42	80	100
Nitrogen content	0.014	0.078	0.136	0.041	0
NaCl	5.47	3.04	1.23	7.59	0
Ash	6.34	3.62	1.35	8.76	0
Water	8.16	43.42	55.3	3.6	0

Table S2. Elemental composition of crude glycerol from different sources [1].

Crude Glycerol	The content of elements (mg/kg)							
	Cu	Mg	Fe	Zn	K	Na	Cl	Ca
**G1**	0.39 ± 0.04	22.55 ± 1.02	5.91 ± 0.20	1.41 ± 0.13	65.89 ± 4.88	23120.03 ± 764.10	33200 ± 425.56	132.21 ± 5.45
**G2**	0.12 ± 0.08	15.26 ± 0.45	2.31 ± 0.68	1.13 ± 0.34	72.74 ± 8.98	13102.66 ± 519.93	18500 ± 30.00	97.06 ± 9.44
**G3**	0.03 ± 0.01	5.305 ± 1.06	458.38 ± 45.84	1.251 ± 0.25	63.137 ± 6.31	5224.84 ± 261.24	7400 ± 59.26	461.95 ± 46.19
**G4**	0.66 ± 0.08	5.454 ± 0.44	26.42 ± 1.20	1.30 ± 0.40	231.32 ± 4.77	31632.89 ± 521.66	46000 ± 21.20	52.36 ± 10.93

Table S3. Relative abundance (%) of surfactin structural analogues present in standard surfactin (Merck) and surfactin extracts obtained from cultures of *Bacillus subtilis* #309 grown in mineral salts medium (MSM) supplemented with glycerol from different sources. The results represent the mean ± standard deviation of three independent experiments.

Surfactin analogue	Surfactin standard	G1 (Biodiesel)	G2 (Biodiesel)	G3 (Stearin)	G4 (Soap)	G5 (Pure glycerol)
C12 Surfactin	2.9 ± 0.1	1.1 ± 0.0	1.1 ± 0.0	2.0 ± 0.1	1.5 ± 0.0	0.9 ± 0.0
C13 Surfactin	14.4 ± 0.5	10.6 ± 0.1	10.8 ± 0.1	12.5 ± 0.1	15.8 ± 0.1	8.9 ± 0.1
C14 Surfactin	35.9 ± 0.2	54.8 ± 0.1	53.1 ± 0.1	54.3 ± 0.3	42.0 ± 0.3	51.6 ± 0.2
C15 Surfactin	41.8 ± 0.4	26.9 ± 0.1	28.6 ± 0.2	25.2 ± 0.2	34.7 ± 0.2	31.5 ± 0.2
C16 Surfactin	3.0 ± 0.0	3.4 ± 0.1	3.6 ± 0.0	2.8 ± 0.1	2.8 ± 0.0	4.4 ± 0.1
C17 Surfactin	0.1 ± 0.0	1.2 ± 0.0	1.0 ± 0.1	0.8 ± 0.0	0.6 ± 0.0	1.1 ± 0.1

References

1. Dobrowolski, A.; Mituła, P.; Rymowicz, W.; Mirończuk, A.M. Efficient conversion of crude glycerol from various industrial wastes into single cell oil by yeast *Yarrowia lipolytica*. *Bioresour. Technol.* **2016**, *207*, 237–243, doi:10.1016/j.biortech.2016.02.039.
